# Creep Failure Behavior in the Weak Areas of 12Cr1MoV Main Steam Pipe Elbow Utilized in Thermal Power Plants

**DOI:** 10.3390/ma18040812

**Published:** 2025-02-12

**Authors:** Shutao Wang, Renqiang Shi, Jian Wu, Chao Yang, Huan Liu

**Affiliations:** 1 Jiangsu Frontier Electric Power Technology Co., Ltd., Nanjing 211100, China; wst0806@126.com (S.W.); srq1033126900@163.com (R.S.); yangchao-666666@163.com (C.Y.); 2 College of Materials Science and Engineering, Hohai University, Changzhou 213200, China; 221608010044@hhu.edu.cn

**Keywords:** 12Cr1MoV steel, main steam pipe, elbow, creep, microstructure

## Abstract

The main steam pipe elbow is a critical metallic component in thermal power plants. Due to prolonged exposure to high temperatures and pressures, it experiences microstructural degradation and creep damage, thereby affecting its service life. Currently, there is debate regarding the location of the weakest region within the elbow, with uncertainty over whether it lies in the inner arc or neutral plane area. This study investigates the microstructure and creep properties of both the inner arc and neutral surface regions of an elbow that has been in operation for 183,088 h, aiming to identify the actual weak region and explore the underlying creep damage mechanisms. The results indicate that under identical temperature and stress conditions, samples from the neutral plane region exhibit significantly higher creep rates and shorter creep rupture times compared to those from the inner arc region. This suggests that the creep life in the vicinity of the inner surface in the neutral plane is markedly lower than that in the inner arc region. Microstructural analysis before and after creep fracture reveals that key factors influencing the creep performance of 12Cr1MoV elbows include carbide size, precipitation amount and distribution, grain size and morphology, as well as the stability and uniformity of grain orientation. Specifically, the growth of intragranular precipitates, the accumulation and non-uniform distribution of grain boundary carbides, and the non-uniform distribution of grain sizes all contribute to the rapid formation of creep cracks and premature material failure. This study concludes that the weakest region in the elbow is located at the inner surfaces of the neutral plane. Future inspections and life assessments of thermal power plant elbows should therefore focus on this area to enhance the accuracy of life evaluations and ensure the safety of thermal power plants.

## 1. Introduction

Metallic components of thermal power plants operate in a high-temperature and high-pressure environment for extended periods. The damage caused by temperature and stress significantly impacts the mechanical properties and reliability of these components. To ensure the safe operation of the plant, it is essential to regularly inspect the microstructure and evaluate the mechanical properties of the key metallic components to predict their remaining service life. When the lifespan of a component is substantially reduced due to microstructural degradation, timely replacement is necessary.

The main steam pipeline is a critical component in thermal power plants, with elbows being the most common connection components in the pipeline system. Compared to straight pipes, elbows exhibit more complex stress distributions, leading to a higher likelihood of issues arising during long-term service. Existing research on elbows has revealed that unavoidable shape defects occur during their manufacturing process, primarily characterized by uneven wall thickness and section ellipticity. These two types of shape variations significantly influence the actual stress distribution within the elbow, thereby affecting the microstructural evolution of the material during service and weakening its mechanical properties and service life [1,2,3]. Ye et al. [4] utilized a finite element model of an unequal-thickness P92 elbow to analyze the stress concentration regions under working pressure. Their findings revealed that the stress concentration primarily occurred in the inner arc region of the elbow. An and Li et al. [5,6] conducted independent finite element analyses on cases with elliptical and circular cross-sections along the bend arc, revealing a transition in stress concentration zones from the inner arc area to the neutral plane. Due to these geometric factors, existing studies exhibit variability in identifying the weak regions of elbows. Therefore, it is essential to conduct a detailed analysis of the microstructural characteristics of different areas within elbows to accurately determine the location of these weak regions.

12Cr1MoV alloy steel is extensively utilized in the main steam pipes of thermal power plants due to its excellent high-temperature strength and creep resistance. During the operation of these plants, elbows are primarily subjected to damage from creep and fatigue. In particular, under high-temperature and high-pressure conditions, creep damage has a more pronounced effect on metallic materials [7,8,9]. Consequently, the investigation of creep damage is a critical focus in current elbow research. Long-term creep damage in 12Cr1MoV alloy results in the precipitation of Cr, Mo, and V elements from the solid solution, as well as their transformation into carbides, and subsequent aggregation and growth. This process usually leads to severe pearlite spheroidization, which weakens the solid-solution strengthening effect and significantly reduces the thermal strength and creep resistance of 12Cr1MoV low-alloy heat-resistant steel [10]. Tang et al. [11] conducted metallographic analysis and tensile tests at room temperature and at high temperatures to assess the remaining life of a 12Cr1MoV main steam pipe that exhibited spheroidization after 70,000 h of service in a power plant. However, the failure mode under high-temperature tensile testing is characterized by instantaneous fracture, primarily driven by instantaneous plastic deformation and brittle fracture behavior. In contrast, the failure mechanism of creep damage involves long-term cumulative effects such as the formation and propagation of voids and cracks. Therefore, it is evident that short-term tensile tests cannot adequately represent the creep process. In addition, the presence of shape defects in elbows leads to varying stress distributions across different regions under high-temperature and high-pressure service conditions, resulting in differing degrees of creep damage [12,13]. Extensive research indicates that the neutral plane and inner arc regions exhibit the most pronounced stress concentrations. However, there remains controversy regarding the precise location of the weakest area.

Therefore, this study selected a 12Cr1MoV main steam pipeline elbow that has been in service as the research object. Samples were extracted from the inner arc region and neutral plane region of the elbow for microstructural characterization and creep mechanical property evaluation. The differences in microstructure evolution and mechanical properties between these two regions were analyzed and compared. On one hand, this analysis identified the weak areas of the elbow; on the other hand, it explored the distinct creep damage behaviors in these regions.

## 2. Methods

### 2.1. Materials and Creep Testing Methods

**Figure 1:**
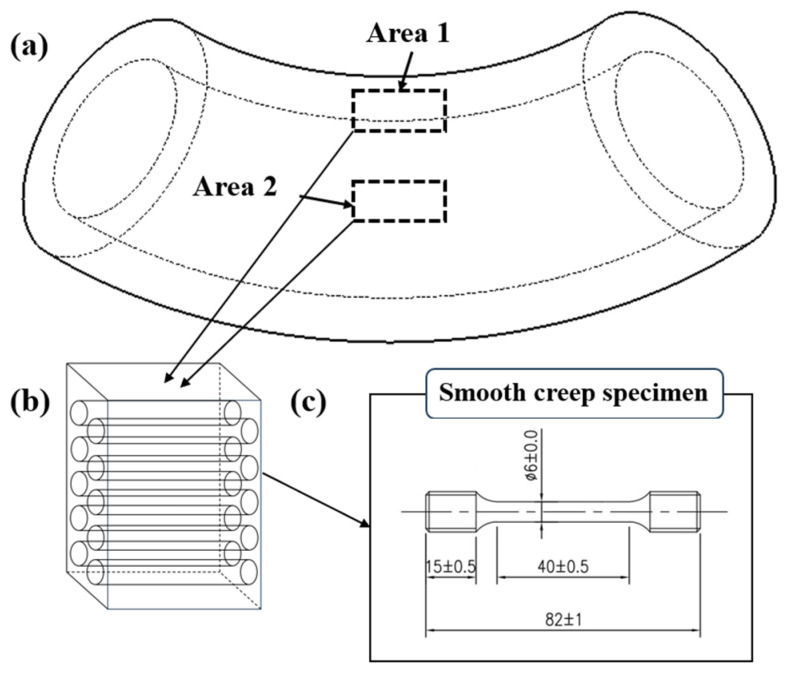
Sampling locations and specifications of the creep test specimens. (**a**) Sampling regions; (**b**) Sampling procedure; (**c**) Dimensions of the creep test specimens (mm).

The investigation object of this study is a 12Cr1MoV steel elbow that has been in service for 183,088 h at a power plant provided by Jiangsu Fangtian Electric Power Co., Ltd., (Nanjing, China). The elbow under investigation was fabricated via hot bending, followed by a heat treatment process that includes normalizing at 980–1020 °C and tempering at 720–760 °C. The shape diagram of the elbow is shown in Figure 1a. The outer diameter and wall thickness are 419 mm and 70 mm, respectively. To investigate the differences in creep performance between weak regions of the elbow, two areas with conflicting findings in the literature were selected for testing. The selection diagram is also shown in Figure 1a. Position 1 corresponds to the inner arc region, while Position 2 corresponds to the neutral plane region. Samples from these two positions are then denoted as Sample 1# and Sample #2, respectively, and both samples were taken from the region near the inner surfaces of the elbow. Creep specimens were cut perpendicular to the wall thickness, as illustrated in Figure 1b. The shape and dimensions of the creep specimens are detailed in Figure 1c, with the gauge length and diameter being 40 mm and 6 mm, respectively. During steady-state operation, the elbow operates under conditions of 540 °C temperature and 15.6 MPa internal pressure. To accelerate the creep process, the test temperature was set to the service temperature, and stress levels of 120 MPa, 130 MPa, and 140 MPa were applied. The creep testing was conducted using an RDL50 electronic persistent creep testing machine. The detailed steps for the creep test are as follows: (1) Secure the sample in the testing machine; (2) Increase the temperature to 540 °C while applying a preload of 200 N (the heating process takes approximately 1 h); (3) Maintain the temperature at 540 °C for 1 h, and then apply the set stress to initiate the experiment.

### 2.2. Microstructural Characterization

The microstructure of the samples before and after the creep test was characterized by optical microscopy (OM, VMM4000R, MEGA Instrument, Suzhou, China), scanning electron microscopy (SEM, Zeiss Gemini 300, Carl Zeiss AG, Oberkochen, Baden-Württemberg, Germany), electron backscatter diffraction (EBSD) analysis, and transmission electron microscopy (TEM, Tecnai G2, FEI, Hillsboro, OR, USA). For metallographic and SEM samples, the specimens were sequentially polished using metallographic sandpapers from 400 grit to 2500 grit, followed by mechanical polishing on a metallographic polisher. During this process, a diamond spray polishing agent with a particle size of 2.5 μm was used, and the samples were subsequently etched for 10 s in a 4 wt.% nitric acid-alcohol solution. EBSD samples underwent the same initial polishing treatment as metallographic samples and were then electropolished using a 5% perchloric acid solution. The EBSD instrument used is an Oxford Symmetry system, with imaging parameters set at 20 kV voltage and a step size of 250 nm. TEM samples were mechanically thinned to a thickness of 50–90 μm using 400–2000 grit sandpapers and were finally perforated through ion milling.

**Figure 2:**
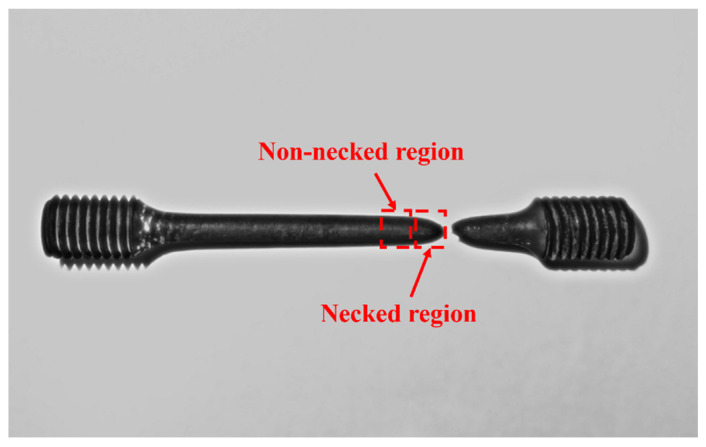
Photograph of the selected necked and non-necked regions of the fractured creep test specimens.

SEM observations were conducted on the fracture surface of the specimens after creep testing. Additionally, the longitudinal cross-sections of the samples were also examined, with the sampling locations as indicated in Figure 2. Samples approximately 4 mm in length were extracted from both the necked and non-necked regions adjacent to the creep fracture site for detailed examination. The observation plane was oriented parallel to the tensile direction of the creep test.

## 3. Results and Discussions

### 3.1. Creep Test Results

**Figure 3:**
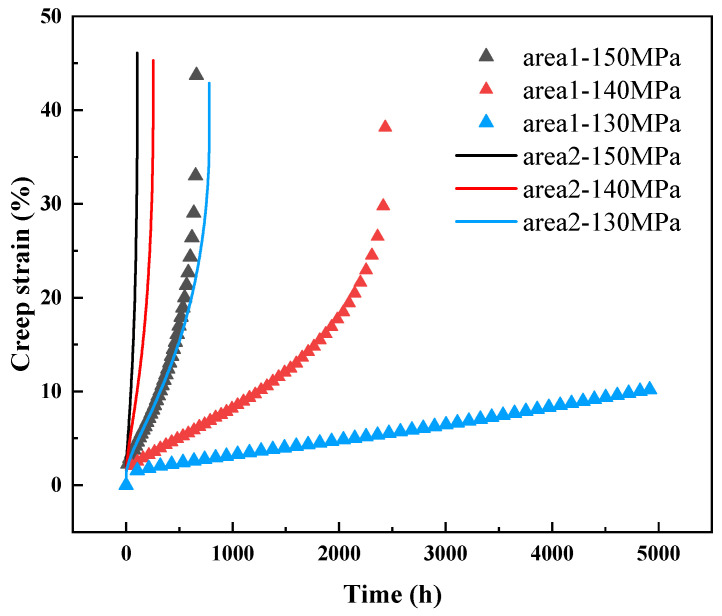
Creep curves of samples 1# and 2# under different stress conditions.

The typical creep test curves for the samples from regions 1# and 2# of the elbow are presented in Figure 3. Table 1 summarizes the corresponding creep rupture times, steady-state creep rates, and steady-state creep durations. It is evident that under both high and low stress conditions, sample 2# exhibits a significantly higher creep rate compared to sample 1#, along with a markedly shorter creep rupture time. Specifically, at 140 MPa, the difference in creep rupture time between the two samples is approximately tenfold. Moreover, at 130 MPa, the sample 1# remains unbroken even after nearly 5000 h of testing, indicating that sample 2# demonstrates substantially inferior creep resistance. Furthermore, as illustrated in Figure 3, the creep curves of sample 2# at 140 MPa and 150 MPa exhibit almost no steady-state creep region. Instead, the samples rapidly transition into the accelerated creep stage and subsequently fail. In contrast, sample 1# displays a more pronounced steady-state creep stage, with its steady-state creep rate being at least one order of magnitude lower than that of sample 2# under identical conditions. This observation further confirms that the creep resistance of the neutral plane region corresponding to sample 2# is significantly inferior to that of the inner arc region represented by sample 1#. To elucidate the reasons for the marked deterioration in the creep resistance of region 2, comprehensive characterization and analysis of the creep test specimens are required. Consequently, a series of characterizations were performed on the fractured creep samples.

**Figure 4:**
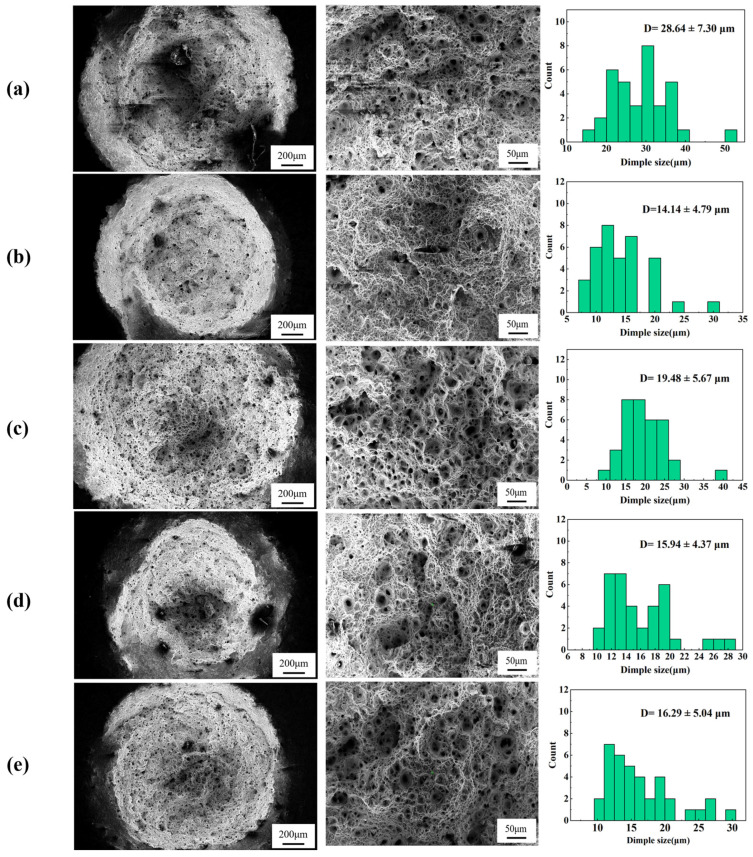
Comparison of fracture cross-sectional morphologies at low and high magnifications, along with dimple size measurements for creep test specimens. (**a**) Sample 1# at 150 °C; (**b**) Sample 2# at 150 °C; (**c**) Sample 1# at 140 °C; (**d**) Sample 2# at 140 °C; (**e**) Sample 2# at 130 °C.

Figure 4 illustrates the fracture morphology of the creep samples after failure and the size distribution of dimples on the fracture surfaces. Additionally, the lengths and areas of characteristic regions on the fracture, such as shear lips and fiber zones, were statistically analyzed and summarized in Table 2. From the low-magnification SEM images, it is evident that under identical conditions, the fracture cross-sectional dimensions of sample 1# are significantly larger than those of sample 2#. This suggests that during long-term creep, sample 1# can more effectively distribute the applied load, thereby reducing the creep rate and enhancing creep performance [14]. Furthermore, the average dimple sizes from the creep test samples shown in Figure 3 indicate that under the same conditions, sample 1# exhibits larger average dimple sizes compared to sample 2#. Generally, larger and deeper dimples signify better material plasticity. This observation implies that during the creep process, sample 1# can mitigate stress concentrations through plastic deformation, delaying fracture initiation and resulting in the formation of more extensive large dimples.

A greater area and higher proportion of the fiber zone indicate a higher concentration of strengthening phases or tougher regions within the material. This characteristic facilitates stress relief, enhances energy absorption during crack propagation, and delays fracture initiation. Moreover, under prolonged exposure to high temperatures and sustained stress, the material can mitigate creep through plastic deformation in the fiber zones [15]. Comparing Figure 4 with the data from Table 2 further corroborates this observation. Specifically, at both 140 MPa and 150 MPa, the fiber area proportion of Sample 1# is significantly larger than that of Sample 2#.

The shear lip primarily forms in the late stages of fracture, representing the final shear region developed under shear stress due to the reduced effective cross-sectional area caused by crack propagation. The length of the shear lip reflects the extent of the shear zone during material fracture. A longer shear lip and a higher proportion of shear lip length typically indicate premature instability and extensive plastic deformation in the material. Consequently, materials with superior creep resistance can distribute stress more uniformly, prevent excessive localized stress concentrations, and maintain a low deformation rate over extended periods, thereby reducing the formation of shear lips [16]. Seen from Figure 4 and Table 2, it is evident that at both 140 MPa and 150 MPa, the shear lip length ratio of Sample 2# is significantly greater than that of Sample 1#. This suggests that Sample 2# exhibits markedly inferior creep resistance compared to Sample 1#, leading to larger local stress concentration and faster fracture under creep conditions. The aforementioned fracture analysis further confirms substantial differences in creep resistance between the two samples, and the underlying reasons for these differences require further investigation through microstructural analysis.

### 3.2. Microstructure of Samples Before and After Creep Testing

#### 3.2.1. Microstructure of Samples Before Creep Testing

**Figure 5:**
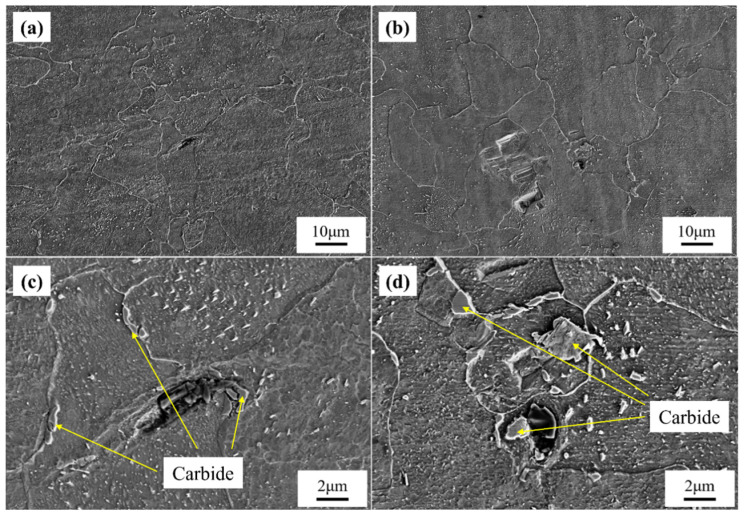
SEM images of the 1# and 2# samples before creep testing at low and high magnifications. (**a**,**c**) Specimen 1; (**b**,**d**) Specimen 2.

To conduct a comparative microstructural analysis with the samples after creep testing, SEM observations were performed on the original microstructures of Samples 1# and 2# from the 12Cr1MoV elbow, as shown in Figure 5. The overall microstructure morphology of both samples is similar. However, after prolonged service, both samples exhibited some creep cavities, with Sample 2# showing more pronounced and extensive micro-damage. High-magnification SEM images reveal that carbides formed near the grain boundaries in both samples. In Sample 1#, the carbides are relatively small, whereas the carbides have noticeably coalesced into larger aggregates in Sample 2#, indicating more severe microstructural degradation during service. Additionally, the characteristics of the cavities suggest that the coalescence and growth of carbides promote the formation of creep cavities, which can further develop into creep cracks. Although some creep cracks also formed in Sample 1#, they did not progress to form larger cavities as observed in Sample 2#.

**Figure 6:**
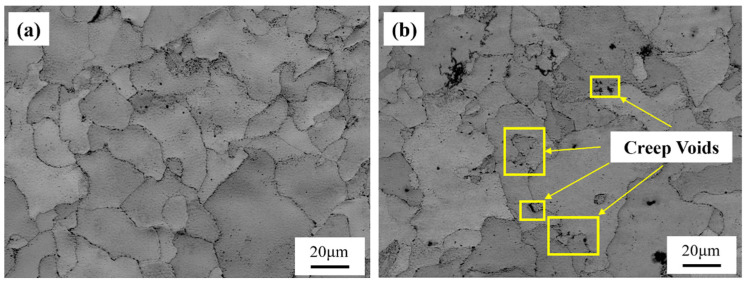
BC images of the microstructure of Specimens (**a**) 1 and (**b**) 2 without creep testing.

**Figure 7:**
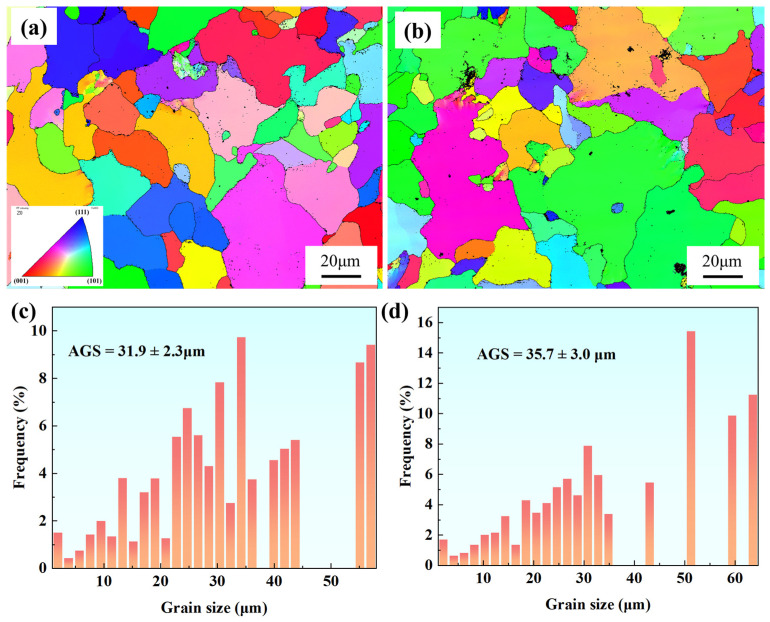
IPF maps and grain size distributions of Samples 1# (**a**,**c**) and 2# (**b**,**d**). The colors in (**a**,**b**) represent grains of different orientations as illustrated in inset of (**a**). The bars in (**c**,**d**) represent the frequency of occurrence of the specific grain size.

Figure 6 presents the BC diagrams of Samples 1# and 2#. It is evident that Sample 1# exhibits a more complete grain structure with better grain boundary stability, resulting in fewer and smaller creep cavities. In contrast, Sample 2# shows irregular grain morphology with a higher density of creep cavities distributed along the grain boundaries. This indicates that under identical service conditions, Sample 2# is more susceptible to stress concentration and micro-damage, leading to performance degradation. Figure 7 shows the inverse pole figure (IPF) maps and the average grain sizes of two samples. The IPF images reveal that the grain distribution in Sample 1# is relatively uniform with well-defined grain shapes, whereas Sample 2# displays irregular grains with significant size variations. The average grain size of Sample 1# is 31.9 ± 2.3 μm, while that of Sample 2# is 35.7 ± 3.0 μm. Sample 1# has a slightly smaller average grain size with a more concentrated distribution, where most grains range between 20 and 40 μm. Conversely, Sample 2# exhibits larger grains with a less uniform distribution, and some grains exceed 50 μm in size.

**Figure 8:**
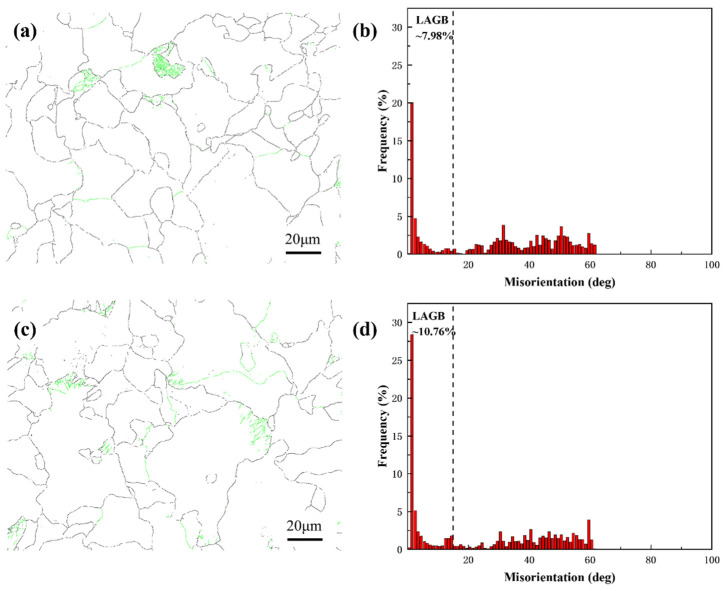
Grain boundary maps and corresponding grain boundary misorientation distribution plots of Samples 1# (**a**,**b**) and 2# (**c**,**d**). The green lines in (**a**,**c**) represent low-angle grain boundaries, and the black lines represent high-angle grain boundaries. Red bars in (**b**,**d**) represent the frequency of occurrence of the specific misorientation angle.

Figure 8 presents the grain boundary maps and corresponding grain boundary misorientation distributions of the two samples. It can be observed that the proportion of low-angle grain boundaries (LAGBs) in Sample 1# is 7.98%, which is relatively low overall. High-angle grain boundaries (HAGBs) dominated the microstructure, displaying broad misorientation distributions between 15° and 60°. In contrast, Sample 2# exhibited an elevated LAGB fraction (10.76%, an increase of 2.78%), with HAGBs showing a narrower distribution, suggesting incomplete recrystallization. Sample 1# showed evidence of complete recrystallization with stable grain morphology, while Sample 2# retained a higher proportion of LAGBs and restricted HAGB distribution, suggesting persistent substructural features within grains.

**Figure 9:**
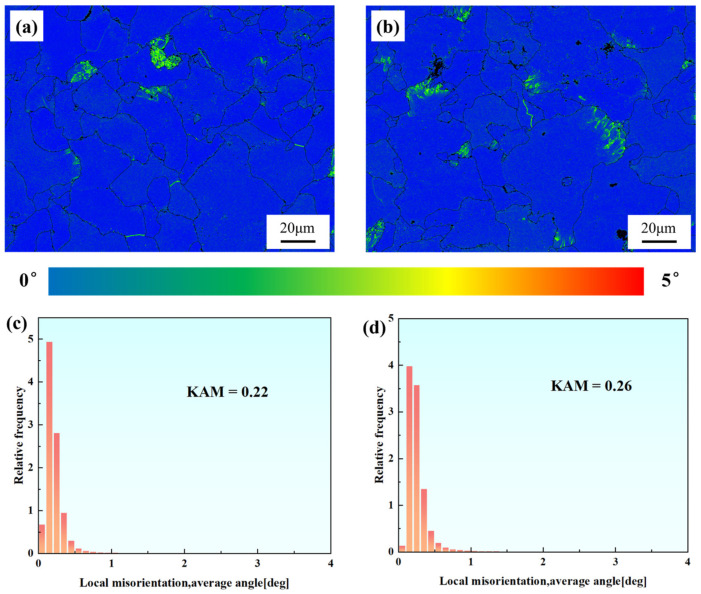
Kernel Average Misorientation maps and distributions of the two samples. (**a**,**b**) Sample 1#; (**c**,**d**) Sample 2#. The color bar represents the misorientation degree.

Figure 9 illustrates the Kernel Average Misorientation (KAM) maps of the two samples. Sample 1# showed predominantly low-angle misorientations (<1°), with a mean KAM value of 0.22°. In contrast, Sample 2# exhibited a higher mean KAM value (0.26°) and displayed a broader misorientation distribution, particularly for angles exceeding 1°, characterized by a more gradually declining columnar distribution. Overall, the dislocation density in both samples is relatively low. However, local strain significantly increases in the grain boundary regions of certain grains, particularly near the locations of creep cavities, indicating a higher density of dislocations and stress concentration in these areas.

Through the aforementioned analysis of the microstructures of Samples 1# and 2#, it is evident that there are significant differences between the two samples in terms of carbide morphology, grain size distribution, grain boundary misorientation distribution, and dislocation density. These observations indicate that Sample 2# exhibits more pronounced microstructural degradation, which may account for the notable differences in their creep properties. In the following section, we will characterize the microstructure of the samples after creep testing and compare them with the pre-creep samples to identify the primary factors contributing to the observed differences in creep performance.

#### 3.2.2. Microstructure of the Samples After Creep Testing

**Figure 10:**
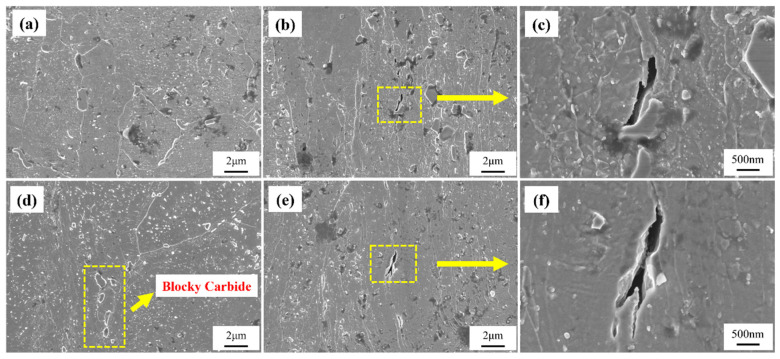
Low- and high-magnification SEM images of the non-necked (**a**,**d**) and necked regions (**b**,**c**,**e**,**f**) of Specimens 1 and 2.

Due to the significant difference in creep behaviors between the two samples under the test condition of 140 MPa, the microstructure after creep testing under this condition was selected for further characterization. Figure 10 shows the SEM microstructures of the non-necked and necked sections of both samples. It can be observed that after the creep test, the grain boundaries in both samples became relatively indistinct, which is typically attributed to increased creep damage and grain boundary slip, leading to irregular grain morphology [17,18]. Additionally, in the non-necked section, carbide precipitation in Sample 2# extensively filled the grains, with some carbides aggregating and growing into blocky structures. Although Sample 1# also exhibited some blocky carbides after prolonged creep testing, its overall grain structure remained relatively clean. The uneven distribution or excessive accumulation of carbides at grain boundaries can lead to grain boundary embrittlement, thereby weakening the overall creep resistance of the metallic material [19,20]. This suggests that after prolonged service, the composition and microstructure of Sample 2# are more heterogeneous, making it more susceptible to stress concentration near grain boundaries and second-phase particles during creep testing. Consequently, this affects the overall creep resistance of the material and leads to premature fracture. In the necked region, Sample 2# exhibits extensive formation of large blocky carbides near the grain boundaries, which was not observed in Sample 1#. This difference arises because carbide size has a significant impact on creep properties. Large blocky carbides exhibit higher stability and, when uniformly distributed near grain boundaries, enhance creep resistance through the pinning effect, which inhibits grain boundary sliding. Conversely, small carbides have lower stability due to their smaller size and can cause internal stress accumulation during creep, thereby reducing the material’s creep resistance [21,22].

Based on the high-magnification crack morphology, it can be observed that Sample 2# has undergone only 253 h of creep testing, yet the creep cracks formed by the accumulation of creep cavities are larger than those in Sample 1#, which was subjected to 2434 h of creep. This indicates that within a short period, Sample 2# has developed a higher density of creep cavities, which have coalesced into larger cracks. The formation of creep cavities is typically not the result of a single factor but rather a synergistic effect of multiple factors. This suggests that after service, Sample 2# exhibits greater overall brittleness, promoting the formation and rapid growth of creep cavities. Moreover, the uneven distribution of carbides in Sample 2# facilitates the rapid accumulation of cavities, which then expand rapidly under local stress concentration, leading to crack formation.

**Figure 11:**
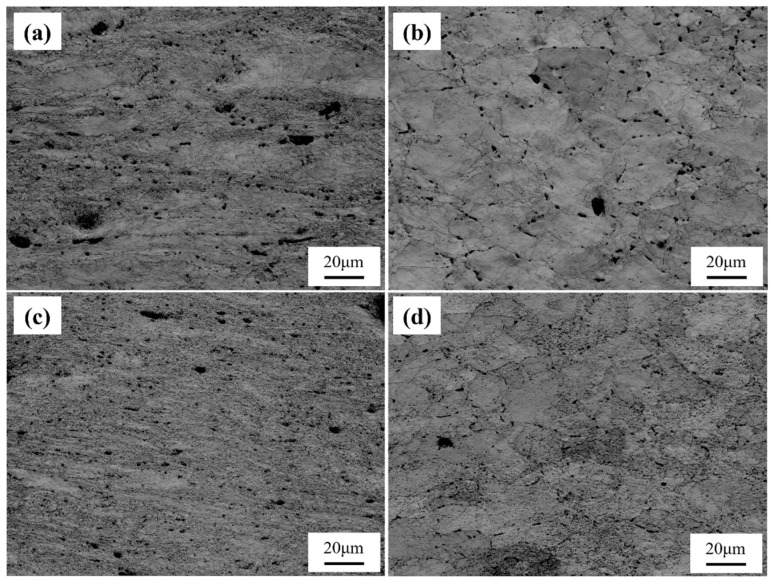
Creep cavity distributions in the necked and non-necked regions of Specimens 1 and 2. (**a**) Necked region of Specimen 1; (**b**) Non-necked region of Specimen 1; (**c**) Necked region of Specimen 2; (**d**) Non-necked region of Specimen 2.

Figure 11 shows the band contrast (BC) maps of the non-necked and necked sections of the two samples, from which creep cavities can be clearly observed. In the necked section, Sample 1# exhibits a higher density of large creep cavities, with noticeable depressions around some of the larger cavities. Comparing this with the fracture morphology in Figure 4, it is evident that Sample 1# forms more extensive and coarser cavities, indicating its higher capacity to accommodate cavities, leading to the formation of deeper dimples. Conversely, the creep cavities in the necked section of Sample 2# are significantly smaller, suggesting a limited capacity for cavity accommodation. Additionally, in the non-necked section, the grain boundaries of Sample 1# are more distinct, but more cavities appear in the grain boundary regions. However, unlike those in the necked section, these cavities have flush surroundings, possibly due to the lack of second-phase particle aggregation in the grain boundary region of Sample 1#. During EBSD sample preparation, these dispersed particles are more likely to be polished away, leaving behind cavity-like features. Although fewer cavities are observed in the non-necked section of Sample 2#, the grain boundary morphology is less distinct, and the aggregation of second-phase particles is clearly visible.

**Figure 12:**
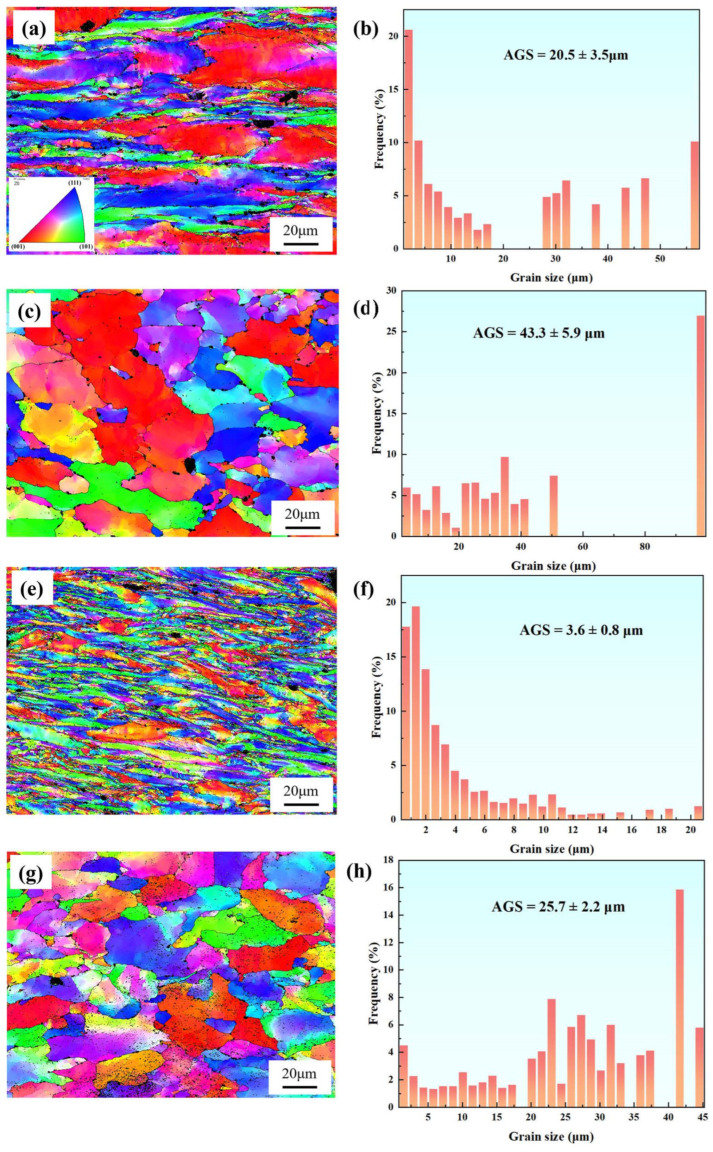
IPF maps (**a**,**c**,**e**,**g**) and grain size distributions (**b**,**d**,**f**,**h**) in the necked and non-necked regions of Specimens 1 and 2. (**a**,**b**) Necked region of Specimen 1; (**c**,**d**) Non-necked region of Specimen 1; (**e**,**f**) Necked region of Specimen 2; (**g**,**h**) Non-necked region of Specimen 2.

Figure 12 shows the IPF maps and grain size distributions of the necked and non-necked regions in two samples. The grain sizes of both the necked and non-necked sections of Sample 2# are smaller than those of Sample 1#, with the grain size in the necked section being only one-sixth that of Sample 1#. This indicates that during the creep process, dislocation slip in Sample 1# is more stable, allowing it to better adapt to external stress and effectively disperse stress, thereby enhancing its creep resistance. In contrast, Sample 2# experiences significant stress concentration during creep, leading to pronounced grain refinement and elongation. Generally, small grains enhance material strength through grain boundary strengthening and dislocation obstruction (Hall–Petch effect). However, excessively fine grains can result in reduced plasticity, increasing material brittleness under stress and thus diminishing high-temperature fracture resistance. During high-temperature creep, finer grains may lack sufficient deformable regions, causing pores to concentrate at local stress concentration points and leading to premature brittle fracture [23,24,25,26]. Notably, during necking, the grain size of Sample 2# decreases dramatically, further indicating that localized stress concentration intensifies grain deformation and promotes significant grain reorientation. Conversely, the grain orientation of Sample 1# remains relatively stable throughout the long-term creep process, demonstrating superior microstructure stability.

**Figure 13:**
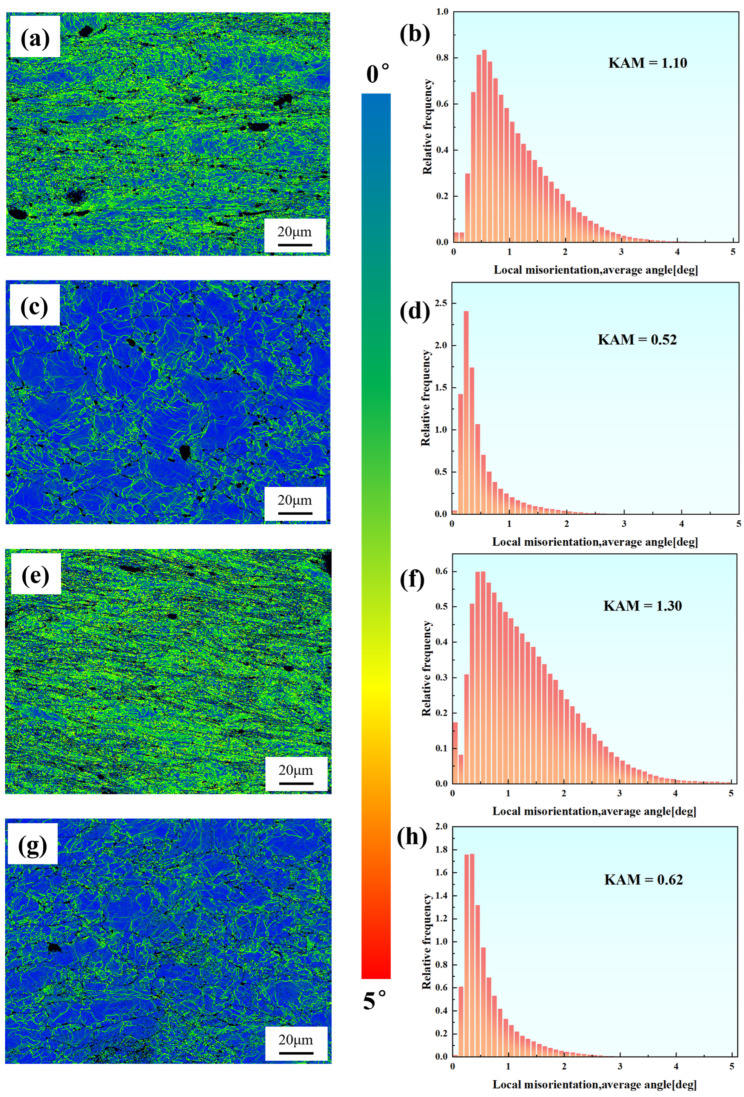
KAM maps of the necked and non-necked regions of Specimens 1 and 2. (**a**,**b**) Necked region of Specimen 1; (**c**,**d**) Non-necked region of Specimen 1; (**e**,**f**) Necked region of Specimen 2; (**g**,**h**) Non-necked region of Specimen 2. The color bar represents the misorientation degree.

Figure 13 shows the KAM maps of different regions of the two samples. Localized stress concentration in Sample 2# accelerated creep deformation, resulting in elevated dislocation densities compared to Specimen 1 in both necked and non-necked regions. The extensive grain refinement observed in the necked region of Sample 2# suggests partial dislocation annihilation through dynamic recrystallization, implying that the total dislocation generation in Specimen 2 significantly exceeded that of Specimen 1 during creep.

**Figure 14:**
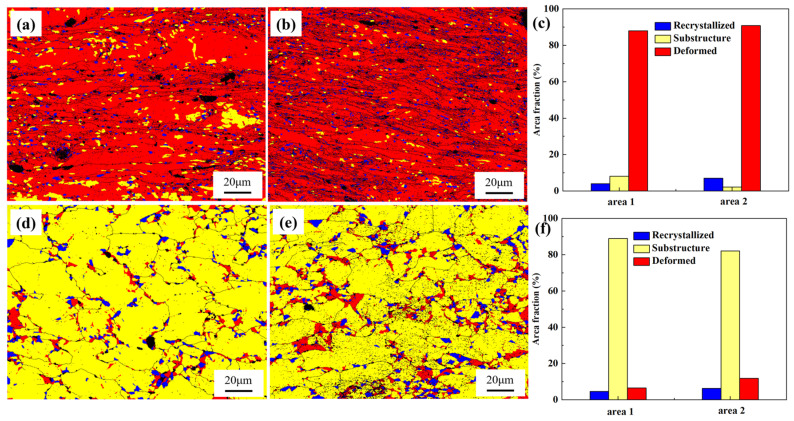
Recrystallization distribution variations in the necked and non-necked regions of Specimens 1 and 2. (**a**) Necked region of Specimen 1; (**b**) Necked region of Specimen 2; (**c**) Comparison of recrystallization distributions in the necked regions of Specimens 1 and 2; (**d**) Non-necked region of Specimen 1; (**e**) Non-necked region of Specimen 2; (**f**) Comparison of recrystallization distributions in the non-necked regions of Specimens 1 and 2.

Recrystallization analysis (Figure 14) showed that the proportion of substructures in the necked section of Sample 1# is significantly higher than that of Sample 2#, and it is observed that these substructures cluster to form lamellar structures. Generally, when materials undergo prolonged creep or plastic deformation, stress tends to localize in certain regions, promoting the formation of lamellar substructures in these areas. Since grain boundaries typically act as barriers to dislocation movement, the aggregation of substructures can enhance grain boundary stability, preventing grain boundary slip and crack propagation, thereby improving the material’s creep resistance. Additionally, both in the necked and non-necked sections, the proportion of deformed grains in Sample 2# is higher than in Sample 1#. This indicates that Sample 2# experienced greater stress over a shorter period, leading to more severe grain deformation in the non-necked region compared to Sample 1#.

**Figure 15:**
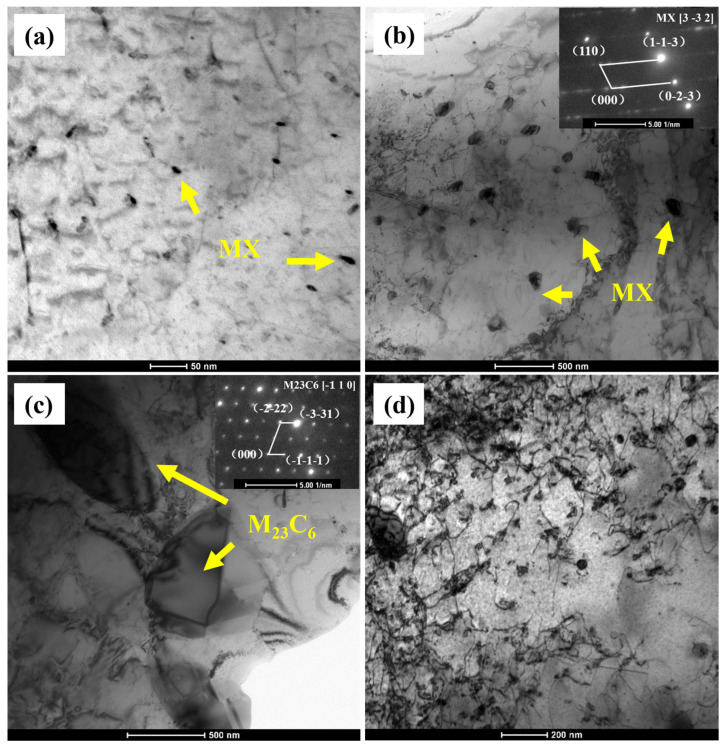
TEM micrographs of the microstructure of Specimens 1 and 2. (**a**) Necked region of Specimen 1; (**b**–**d**) Necked region of Specimen 2.

Figure 15 shows the TEM observations of the necking regions in Samples 1 and 2#. MX precipitates are observed in the matrix of both alloys. In Sample 1#, the MX phase particles are uniformly distributed and small in size (approximately 5 nm), existing in a dispersed form within the matrix. This uniform dispersion enhances the material’s creep resistance by effectively pinning dislocations and inhibiting their movement, thereby reducing creep deformation. Additionally, the interaction between the MX phase and dislocation lines is evident, further supporting this effect. In contrast, the MX phase particles in Sample 2# exhibit non-uniform distribution and local aggregation, with significantly larger particle sizes (approximately 100 nm). These aggregated MX particles are predominantly concentrated near grain boundaries, potentially weakening the grain boundaries. The large MX particles have a diminished pinning effect on dislocations, and the aggregation at grain boundaries may induce grain boundary slip and stress concentration. Consequently, dislocation slip is less effectively pinned, leading to rapid formation and propagation of creep cavities. Overall, the fine and uniformly dispersed MX phase in Sample 1# significantly enhances the matrix strength, while the oversized particles in Sample 2# weaken this strengthening effect.

Moreover, micron-sized MX phases originally present in the microstructure (as shown in Figure 15c) exhibit irregular shapes and sharp interfaces, which can lead to stress concentration during creep. This makes Sample 2# more susceptible to interface slip and cavity expansion, ultimately accelerating early material failure. Additionally, in the necked region of Sample 2# (Figure 15d), a dense distribution of intragranular dislocations is observed, indicating significant dislocation accumulation during necking. This high dislocation density suggests intensified grain boundary slip and dislocation climb, concentrating deformation during the creep process. In contrast, no significant dislocation aggregation is observed in the necked region of Sample 1#.

### 3.3. Key Factors Influencing the Creep Performance of Different Regions of the Elbow

From the above results, it is evident that despite the neutral plane region of Sample 2# and the inner arc region of Sample 1# being subjected to the same service environment, their performance exhibits significant differences. This clearly indicates that the degree of damage experienced by different regions of the elbow during steady-state operation of a plant is not uniform.

Based on the microstructure analysis results presented above, the most significant factors influencing the creep resistance of 12Cr1MoV elbows are identified as follows:

(1) Carbide Morphology. Microstructure analysis of samples 1# and 2# reveals that carbides significantly influence the creep properties of the material. The size, precipitation amount, and distribution uniformity of carbides all play crucial roles in determining the material’s creep resistance. When carbides are distributed within the grains, the precipitation of nanoscale carbides enhances the matrix’s creep resistance. However, as these nanoscale carbides grow, their effectiveness diminishes, leading to a decrease in creep resistance. When carbides are located near grain boundaries, larger carbides tend to have a positive effect on creep performance due to their higher stability. Uniformly distributed large carbides near grain boundaries can significantly strengthen the material’s creep resistance. Conversely, small carbides exhibit poor stability due to their smaller particle size. While relatively uniform distributions of small carbides may have minimal impact on creep resistance, excessive and non-uniform distributions can induce stress concentration, accelerating creep fracture [23,24,25,26].

Furthermore, the volume fraction of carbide precipitates influences creep resistance. At low precipitation volumes, the effect on creep resistance is negligible. However, at higher volume fractions, uniformly distributed carbides may coalesce to form larger blocky carbides, paradoxically enhancing material stability. In contrast, irregular distributions can generate stress concentrations, promoting the nucleation and evolution of creep cavities and cracks, thereby degrading creep resistance [27,28].

(2) Grain Size and Morphology. EBSD analysis showed that larger grains can enhance creep resistance by facilitating stress distribution during deformation, contrary to conventional expectations. Conversely, excessive grain refinement can localize deformation, promoting cavity concentration and reducing ductility [29,30]. Grain uniformity significantly influences creep resistance. Sample 2# exhibits a dispersed distribution with a mixture of large and small grains prior to creep, which can lead to uneven stress distribution during the creep process.

(3) Grain Orientation. The impact of grain orientation on creep resistance is relatively straightforward. Under identical creep conditions, increased material deformation leads to changes in grain orientation and an increase in orientation difference. Consequently, samples with more stable and uniform grain orientations exhibit superior creep resistance and extended creep life during prolonged creep processes [31,32,33].

From the analysis of various influencing factors and microstructural characteristics, it is evident that stress concentration in the neutral plane region results in greater damage compared to the inner arc region. However, the experimental results presented here preliminarily challenge this assumption. Specifically, within the same service period, the neutral plane region experiences greater damage compared to the inner arc region, leading to a more pronounced performance decline. Existing studies [4] have generally assumed that the inner arc region is the primary stress concentration area and should therefore experience more damage. This assumption is primarily based on finite element analyses, with limited experimental validation [4,5,6]. Moreover, the location of the highest stress in an elbow is influenced by its geometry. Unavoidable shape defects such as uneven wall thickness and section ellipticity can occur during the manufacturing process, particularly in hot bending. We examined the geometry of this specific elbow and found that both types of defects were present. Therefore, this finding strongly suggests that the neutral plane region, rather than the inner arc region, experiences relatively higher stress concentration in actual elbows.

In typical thermal power plants, non-destructive testing (NDT) primarily focuses on the inner arc area of elbows, with the service life of the elbow being assessed based on the microstructure of this region. Consequently, such an approach can lead to inaccurate pipeline life assessments, potentially resulting in severe consequences such as tube rupture. Therefore, future inspections should prioritize the neutral plane region to ensure more accurate evaluations of elbow integrity.

## 4. Conclusions

In this study, the creep behavior of the inner surfaces in both the inner arc region and the neutral surface region of a 12Cr1MoV elbow that has been in service for 183,088 h is compared and analyzed. Fracture analysis and microstructural characterization before and after creep are conducted. Under the same temperature and stress conditions, samples from the neutral plane region exhibit significantly higher creep rates and shorter creep rupture times compared to those from the inner arc region. This suggests that the creep life of the inner surface region of the neutral plane is markedly lower than that of the inner arc region. Microstructural analysis before and after creep fracture reveals that key factors influencing the creep performance of 12Cr1MoV elbows include carbide size, precipitation amount and distribution, grain size and morphology, as well as the stability and uniformity of grain orientation. Specifically, the growth of intragranular precipitates, the accumulation and non-uniform distribution of grain boundary carbides, and the non-uniform distribution of grain sizes all contribute to the rapid formation of creep cracks and premature material failure.

This study concludes that the weakest region in the elbow is located at the inner surface of the neutral plane. This finding holds significant research value and practical implications for accurately assessing the remaining life of elbows in thermal power plants. Future inspections and life assessments of thermal power unit elbows should therefore focus on this area to enhance the accuracy of life evaluations and ensure the safety of thermal power plants.

## Figures and Tables

**Table 1:** Creep test results of the two samples.

Specimen Labeling	Test Temperature/°C	Test Stress/MPa	Creep Rupture Time/h	Steady-State Creep Rate/s^−1^	Steady-State Creep Time/h
1	540	150	661	3.27 × 10^−5^	533.3
		140	2434	7.18 × 10^−6^	1945.5
		130	Unfractured (4917)	5.53 × 10^−7^	Unfractured
2		150	104	2.05 × 10^−4^	83.0
		140	253	7.96 × 10^−5^	202.2
		130	782	2.42 × 10^−5^	630.2

**Table 2:** Data analysis of fracture cross-section SEM morphologies.

Sample	1#-150	2#-150	1#-140	2#-140	2#-130
Shear Lip Length (µm)	343.2	354.5	354.1	401.7	285.5
Total Fracture Surface Length (µm)	2038.3	1799.5	2385.5	1815.6	1906.0
Proportion of Shear Lip Length (%)	16.84%	19.70%	14.84%	22.12%	14.98%
Fibrous Zone Area (mm^2^)	1.58	1.16	2.23	1.08	1.56
Total Fracture Surface Area (mm^2^)	3.00	2.53	3.68	2.75	2.92
Proportion of Fibrous Zone Area (%)	52.67%	45.85%	60.60%	39.27%	53.42%

## Data Availability

The original contributions presented in the study are included in the article, further inquiries can be directed to the corresponding author.
